# Impact of the Project P.A.T.H.S. in the Junior Secondary School Years: Objective Outcome Evaluation Based on Eight Waves of Longitudinal Data

**DOI:** 10.1100/2012/170345

**Published:** 2012-04-24

**Authors:** Daniel T. L. Shek, Cecilia M. S. Ma

**Affiliations:** ^1^Department of Applied Social Sciences, The Hong Kong Polytechnic University, Hunghom, Hong Kong; ^2^Public Policy Research Institute, The Hong Kong Polytechnic University, Hong Kong; ^3^Department of Social Work, East China Normal University, Shanghai 200241, China; ^4^Kiang Wu Nursing College of Macau, Macau; ^5^Division of Adolescent Medicine, Department of Pediatrics, Kentucky Children's Hospital, University of Kentucky College of Medicine, Lexington, KY 40536, USA

## Abstract

To assess the effectiveness of the Tier 1 Program of the Project P.A.T.H.S., a randomized group trial with eight waves of data collected was carried out. At the fifth year of data collection, 19 experimental schools (*n* = 2, 662 students) and 24 control schools (*n* = 3, 272 students) participated in the study. Analyses based on individual growth curve modeling showed that participants in the experimental schools displayed better positive youth development than did participants in the control schools in terms of different indicators derived from the Chinese Positive Youth Development Scale, including moral competence and behavioral competence and cognitive behavioral competencies. Significant results were also found when examining the trajectories of psychological development among control and experimental participants who perceived the program to be beneficial. Findings based on longitudinal objective outcome evaluation strongly suggest that the Project P.A.T.H.S. is effective in promoting positive development in Hong Kong secondary school students.

## 1. Introduction

Adolescence is an age of transition. With physical and cognitive maturation taking place in puberty, intrapersonal, and interpersonal changes in adolescents intensify and there are growing social demands and expectations during adolescence. Because of these changes, adolescence is also regarded as an age of stress. The stressors confronting an adolescent might include family stressors (e.g., parental conflict, parental marital problems), interpersonal stressors (e.g., no friends), academic stress (e.g., examination stress), living circumstances-related stressors (e.g., immigration), financial stressors (e.g., poverty), developmental stressors (e.g., early or late maturing), psychological stressors (e.g., lack of life meaning), and social stressors (e.g., high competition). In a Chinese society such as Hong Kong, stress arising from academic excellence and subtle social competition is particularly relevant for Chinese adolescents. Obviously, how to cope with stress in adolescence is an important developmental task for adolescents.

 At the same time, there are several myths about development of adolescents in the Chinese culture [1]. These include as follows (a) young people will grow up automatically, (b) young people are usually troublesome, (c) students in schools with good academic achievement do not have problems, (d) problem free is healthy development, (e) we should focus our attention on the solving of adolescent problems, (f) different adolescent developmental problems require different solutions, and (g) adolescent developmental problems are the sole problem of the government. With reference to these myths, it is argued that (a) adolescent development requires nurturance, (b) young people have strengths and potentials, (c) students attending schools with good academic achievement also display problems, (d) problem free is not fully prepared, (e) solving adolescent problems and prevention are equally important, (f) different adolescent developmental problems have similar root causes and prevention methods, and (g) adolescent development is a topic that is owned by different stakeholders in the society.

 One obvious way to nurture young people is to promote social and emotional competencies of young people [2]. According to the Collaborative for Academic, Social and Emotional Learning (CASEL), “social and emotional learning (SEL) is the process of acquiring the skills to recognize and manage emotions, develop caring and concern for others, make responsible decisions, establish positive relationships, and handle challenging situations effectively. Research has shown that SEL is fundamental to children's social and emotional development—their health, ethical development, citizenship, academic learning, and motivation to achieve. Social and emotional education is a unifying concept for organizing and coordinating school-based programming that focuses on positive youth development, health promotion, prevention of problem behaviors, and student engagement in learning” (http://www.casel.org/). Generally speaking, several SEL attributes are commonly included in different SEL models. These include self-awareness (identifying emotions and recognizing strengths), social awareness (perspective-taking and appreciating diversity), self-management (managing emotions and goal setting), responsible decision making (analyzing situations, assuming personal responsibility, respecting others, and problem solving), and relationship skills (communication, building relationships, negotiation, refusal). Sun and Shek [3] showed that higher level of positive youth development predicted lower level of problem behavior, thus suggesting that positive youth development is an important protective factor in adolescent problem behavior. Emphasis on the importance of SEL is strong in North America and some Asian countries such as Singapore.

 How is SEL implemented in Hong Kong? Instead of focusing on life skills and competencies in students, moral education focusing on values has been emphasized in Hong Kong. While the policy and curriculum guide governing moral education are elegant, there are three problems in the related policy and its actual implementation. First, there are no curriculum materials which have been validated, although there is a pool of suggested curriculum materials that can be used by teachers. Second, there is a wide variation in the mode of implementation of moral education in schools settings. While some schools may incorporate moral education in the formal curriculum, some may use extracurricular activities such as morning assemblies to implement moral education. Finally, rigorous evaluation of moral education programs is rare, although there are administrative audits and collection of management information by the government. The lack of rigorous evaluation implies that there is no way to understand whether there are changes in outcomes as such moral values of the students because input and output evaluation is simply not adequate.

 Shek and Yu [4] reviewed adolescent prevention and positive youth development programs in Asia which have been evaluated by studies adopting true experimental or quasiexperimental designs. They found that compared to Western societies, the number of validated programs in different Asian communities was terribly low. Also, there were comparatively more programs addressing substance abuse than other mental health problems. Compared to evaluated prevention programs, there were very few positive youth development programs. Finally, there were very few rigorously designed evaluative studies of prevention and positive youth development programs over a long period of time. The lack of adolescent prevention and positive youth development programs in Asia has three implications. First, the findings suggest that we lack evidence-based solutions to adolescent developmental issues. Second, the findings mean that we do not clearly know the benefits and harms of the existing programs. Third, the lack of studies also means that there is no accountability of the workers. Against this background, it is important to conduct more evaluation studies for positive youth development programs in Hong Kong.

The Project P.A.T.H.S. (Positive Adolescent Training through Holistic Social Programs) is a youth enhancement program that attempts to promote holistic youth development in Hong Kong. While the Tier 1 Program is a universal positive youth development program adopting a curricular-based approach for students in Secondary 1 to Secondary 3, the Tier 2 program is designed for students with greater psychosocial needs. As far as objective outcome evaluation is concerned, several studies have showed that students who participated in the project showed better development than those who did not participate. Based on the first two waves collected in a randomized group trial, Shek et al. [5] showed that participants in the experimental schools had significantly higher positive youth development levels than those in the control schools. By using the first four waves of data collected in the first two years of the Full Implementation Phase, analyses based on generalized linear models and linear mixed methods similarly showed that students in the experimental schools generally developed better than those in the control schools [6, 7].

With reference to the first six waves of data in the junior secondary school years (i.e., Secondary 1 to Secondary 3), evaluation findings showed that the Project P.A.T.H.S. was able to promote holistic development in the participants. Individual growth curve analyses showed that participants in the experimental schools displayed better positive youth development than did participants in the control schools based on different indicators derived from the Chinese Positive Youth Development Scale, including positive self-identity, prosocial behavior, and general positive youth development attributes. Differences between experimental and control participants were also found when students joining the Tier 1 Program and perceiving the program to be beneficial were employed as participants of the experimental schools [8]. Similarly, longitudinal analyses showed that the participants in the experimental schools displayed lower levels of substance abuse and delinquent behavior than did the control school students. Participants who regarded the program to be helpful also showed lower levels of problem behavior than did the control school students [9]. Similar patterns of findings were observed with the inclusion of the seventh wave of data for analyses [10].

 To replicate the objective outcome evaluation findings and to examine the long-term effectiveness of the Project P.A.T.H.S. over a period of five years, the Wave 8 data were included in the present study. Essentially, we asked whether the program effect could be sustained over a period of two years after the termination of the project at Secondary 3. In the realm of science, replication plays an important role. As pointed out by Campbell and Stanley [11], “we must increase our time perspective, and recognize that continuous, multiple experimentation is more typical of science than once-and-for-all definitive experiments. The experiments we do today, if successful, will need replication and cross-validation at other times under other conditions before they can become an established part of science, before they can be theoretically interpreted with confidence (p. 3).” As longitudinal evaluation studies using objective outcome indicators are rare in different Chinese context, the present study is a pioneer and ground-breaking addition to the literature.

## 2. Methods

### 2.1. Participants and Procedures

During 2006–2011, a total of 7,846 Secondary 1 students (equivalent to Grade 1) were recruited from 48 schools. Shek and associates [12, 13] described the procedures and criteria for recruiting the initial 24 experimental schools and 24 control schools.

Students were measured at baseline in the fall of 2006 (Wave 1) and then followed longitudinally across waves (Wave 2: Spring 2007; Wave 3: Fall 2007; Wave 4: Spring 2008; Wave 5: Fall 2008; Wave 6: Spring 2009; Wave 7: Spring 2010; Wave 8: Spring 2011). In Year 1 (2006-2007), one school withdrew after Wave 1. In Year 2 (2007-2008), Waves 3 and 4 data were collected from the same cohort, with 20 experimental schools (i.e., three schools withdrew after Wave 2) and 24 control schools. In Year 3 (2008-2009), Waves 5 and 6 data were collected from the same cohort with 19 experimental schools (i.e., one experimental school dropped out after Wave 4) and 24 control schools. A total of 3,820 students completed all 8 waves of the study (49%). In the present study, all data were tested as individual growth curve model allows unequal interval spaced time points and missing data [14]. The number of completed questionnaires collected in each measurement occasion can be seen in Table 1.

At pre- and posttest, the purpose of the study was mentioned, and confidentiality of the collected data was repeatedly emphasized to all students in attendance on the day of testing. Parental and student consent had been obtained prior to data collection. All participants responded to all scales in the questionnaire in a self-administration format. Adequate time was provided for the participants to complete the questionnaire. A trained research assistant was present throughout the administration process.

### 2.2. Instruments

Consistent with the procedures used in Year 1, the participants were invited to respond to a questionnaire that comprised different measures of youth development at pretest (i.e., before the program began) and posttest (i.e., after the program ended). The following measures were used.

#### 2.2.1. Chinese Positive Youth Development Scale (CPYDS)

Based on the analyses conducted in Year 1, the item composition of the 15 subscales of the CPYDS is as follows.

1. Bonding Subscale (six items).
2. Resilience Subscale (six items).
3. Social Competence Subscale (seven items).
4. Emotional Competence Subscale (six items).
5. Cognitive Competence Subscale (six items).
6. Behavioral Competence Subscale (modified five items).
7. Moral Competence Subscale (six items).
8. Self-Determination Subscale (five items).
9. Self-Efficacy Subscale (modified two items).
10. Beliefs in the Future Subscale (modified three items).
11. Clear and Positive Identity Subscale (seven items).
12. Spirituality Subscale (seven items).
13. Prosocial Involvement Subscale (five items).
14. Prosocial Norms Subscale (five items).
15. Recognition for Positive Behavior Subscale (four items).

As mentioned by Shek [1], different composite indices derived from the scale were used to assess positive youth development. First, the mean of the total mean score based on 12 subscales (excluding behavioral competence, self-determination, and prosocial norms) could be used as an overall measure of positive youth development (CPYDS-12). Next, as it can be argued that constructs including spirituality, prosocial norms, prosocial involvement, bonding, and recognition for positive behavior are different from the rest of the scales, a summation of 10 subscales (CPYDS-10) assessing psychosocial competence and strengths was used (i.e., resilience, social competence, emotional competence, cognitive competence, behavioral competence, moral competence, self-determination, self-efficacy, beliefs about the future, and clear and positive identity). Third, based on conceptual analyses of the items, one key item was derived for each domain which resulted in a 15-item key measure (KEY 15). Fourth, based on item analysis, a 36-item measure was derived for each domain (KEY 36). Fifth, based on item analysis, a 7-item measure was derived for behavioral competence and moral competence (CPYDS-2). Lastly, Shek and Ma [15] also showed that the 15 subscales in the CPYDS could be further reduced to four dimensions, including cognitive-behavioral competencies (CBC), prosocial attributes (PA), positive identity (PID) and general positive youth development qualities (GPYDQ). In general, higher scores of these variables suggested better positive youth development. The internal consistency of these measures can be seen in Table 2.

#### 2.2.2. Data Analytic Strategies

Individual growth curve (IGC) is an advanced statistical technique which is conducted to examine “aggregates” of individual curves rather than separate analysis of each individual growth curve [14]. This method models individual change over time, determines the shape of the growth curves, explores systematic differences in change, and examines the effects of covariates (e.g., treatment) on group differences in the initial status and the rate of growth. Previous literature shows that this method is commonly used in the field [16, 17].

IGC is an appropriate approach in studying individual change as it creates a two-level hierarchical model that nested time within individual [18–20]. The Level 1 model refers to the within-person or intraindividual change model (i.e., repeated measurements over time). It focuses on the individual and describes the developmental changes for each individual (i.e., the variation within individual over time). Level 1 model estimates the average within-person initial status and rate of change over time. No predictors are included in this model. The basic linear growth model is as showed below.

Level 1 model:

(1) $$Y_{ij}=\beta_{0j}+\beta_{1j}\left(\text{Time}\right)+e_{ij}.\;$$

In our study, *β*_0_ is the initial status (i.e., Wave 1) of the outcome variable for individual *i*. *β*_1_ is the linear rate of change for individual *i* and *e*_*ij*_ is the residual in the outcome variable for individual *i* at Time *t*. *Y*_*ij*_ is the repeatedly measured of the outcome variable for an individual *i* at Time *t*.

To test a nonlinear individual growth trajectory across time, other higher-order polynomial trends (i.e., quadratic and cubic slopes) can also be included for model testing. This is showed in (2), in which *Time* (i.e., the linear slope,  *β*_1_) remains, while *Time^2^* (i.e., quadratic slope, *β*_2_) and *Time^3^* (i.e., cubic slope, *β*_3_) are added in the model:

(2) $$Y_{ij}=\beta_{0j}+\beta_{1j}\left(\text{Time}\right)+\beta_{2j}\left({\text{Time}}^{2}\right)+\beta_{3j}\left({\text{Time}}^{3}\right)+e_{ij}.$$

The Level 2 model captures whether the rate of change vary across individuals in a systematic way. The growth parameters (i.e., the within-subjects intercepts and slope) of Level 1 are the outcome variables to be predicted by the between-subjects variables at Level 2. At this level (In (3)), an explanatory variable (such as, *group *in the present study) is included to analyze the predictor's effect on interindividual variation of outcome variable. The errors are assumed to be independent and normally distributed and that the variance is equal across individuals [19].

The Level 2 model is

(3) $$\begin{array}{cc} Y_{ij}= & \gamma_{0i}+\gamma_{1i}\left(\text{Time}\right)+\gamma_{2i}\left({\text{Time}}^{2}\right)+\gamma_{3i}\left({\text{Time}}^{3}\right)\\ & +\gamma_{01}\left(\text{group}\right)+\gamma_{11}\left(\text{group}\times \text{Time}\right)\\ & +\gamma_{21}\left(\text{group}\times {\text{Time}}^{2}\right)+\gamma_{31}\left(\text{group}\times {\text{Time}}^{3}\right)\\ & +r_{oi}+r_{1i}+\varepsilon_{ij}. \end{array}$$

In our study, *Y*_*ij*_ is the grand mean for the outcome variable for the whole sample at Time *t*. *γ*_0*i*_ is the initial status of the outcome variable for the whole sample at Time *t*. *γ*_1*i*_ is the linear slope of change relating to the outcome variable for the whole sample at Time *t*. *γ*_2*i*_ is the quadratic slope of change relating to the outcome variable for the whole sample at Time *t*. *γ*_3*i*_ is the cubic slope of change relating to the outcome variable for the whole sample at Time *t*. *γ*_01_, *γ*_11_, *γ*_21_, *γ*_31_, are used to test whether the predictor (i.e., *group*) is associated with the initial status, linear growth, quadratic growth, and cubic growth, respectively. *r*_*oi*_, *r*_1*i*_, and *ε*_*ij*_ are the residual errors that is not explained by Level 2 predictors.

In this study, we tested whether treatment was predictive of students' growth parameters (i.e., initial status, linear change, quadratic change, and cubic change) in several positive youth development indicators across time. In particular, the relationships between these indicators and group were estimated after controlling the effect of gender and initial age. The intercept (i.e., initial status) and linear slope were allowed to vary across individuals. To examine the amount of total variation in the outcome variables that is related to between-individual differences, the intraclass correlation coefficient (ICC) is calculated.

A dummy variable was created (i.e., *group*—control versus experimental groups) as a predictor. Participants in the control group were coded as −1 and those in the experimental group as 1. Two covariates (i.e., gender and initial age) were included when examining the predictive program effect on the outcome variables. *Gender* was coded as −1 = male and 1 = female. Similar coding method for a dichotomous variable was found in previous studies [18, 20]. For the continuous variables, grand mean centering method was generally recommended in order to simplify the interpretation of the results [21]. In our study, the mean age was 12. *Initial age* was then centered by subtracting the mean age, and therefore, the centered initial age was generated.

Following the strategy suggested by Singer and Willet [14], a series of models were tested. These included the following: (a) an unconditional model was tested to calculate the ICC, (b) an unconditional growth model served as a baseline model to explore whether the growth curves are linear or curvilinear, (c) two higher order polynomial models were estimated to determine if the rate of change accelerated or decelerated across time, and (d) a conditional model was formed to investigate whether the predictor was related to the growth parameters (i.e., initial status, linear growth, quadratic growth, and cubic growth). The intercept and linear slope were allowed to vary across individuals. Missing data were handled through likewise deletion.

 To facilitate the interpretations of the significant interaction effects, we plotted prototypical trajectories as suggested by Singer and Willett [14] in order to demonstrate the effect of treatment on the rate of change across time. The step in creating prototypical plots is generally identical to the method of plotting graphs in regression [22]. For each outcome variable, a linear mixed model (LMM) via SPSS with maximum likelihood estimation was conducted. As we focused on the entire model (both fixed and random effects), maximum likelihood (ML) method was used [21]. The procedures for analyzing longitudinal data via SPSS can be seen in Shek and Ma [23].

## 3. Results

**Figure 1:**
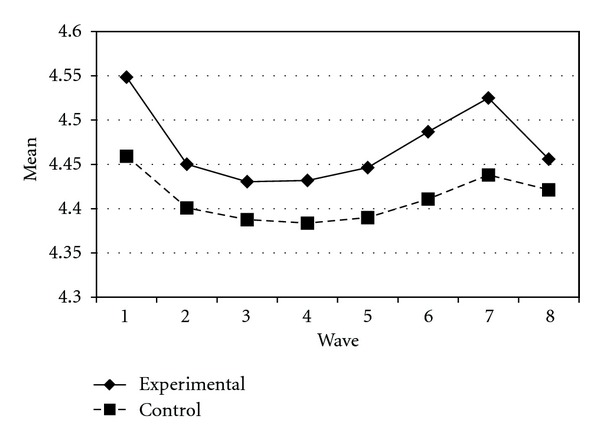
Growth trajectories of the experimental participants and control participants using MC (moral competence subscale) as an outcome indicator.

**Figure 2:**
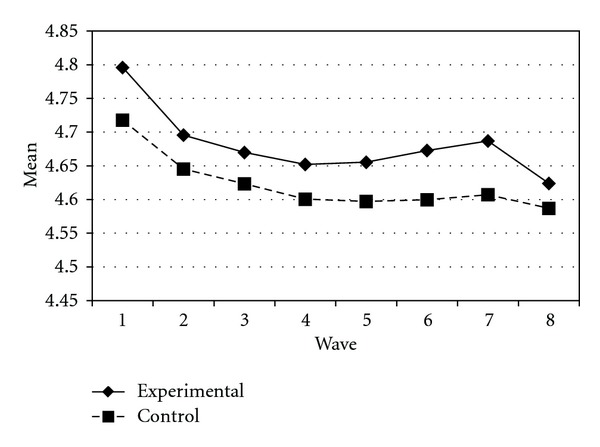
Growth trajectories of the experimental participants and control participants using BC (behavioral competence subscale) as an outcome indicator.

**Figure 3:**
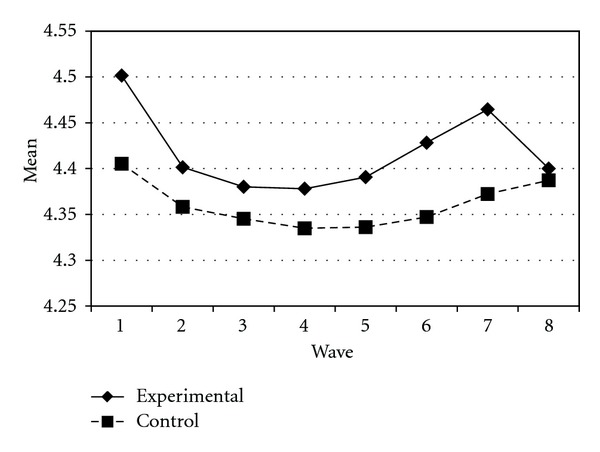
Growth trajectories of the experimental participants and control participants using CPI-1 (one item from clear and positive identity subscale) as an outcome indicator.

**Figure 4:**
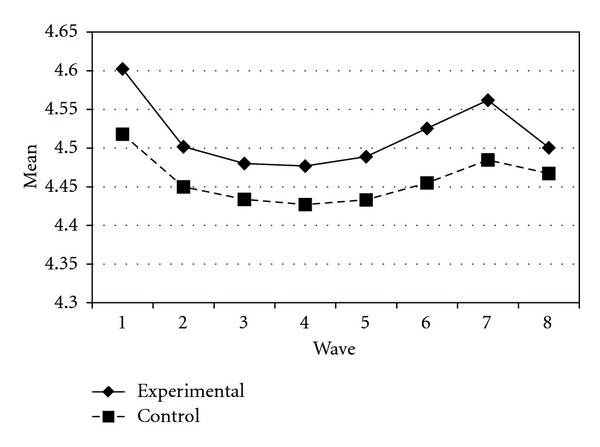
Growth trajectories of the experimental participants and control participants using CPYDS-2 (two subscales (i.e., behavioral competence and moral competence) of the CPYDS) as an outcome indicator.

Tables 3 and 4 present the IGC findings based on several indicators derived from the CPYDS. As can be seen from the tables, there were significant treatment effects across time. Group was a significant predictor of all growth parameters (i.e., the initial status, linear, quadratic, and cubic slopes) in three outcome variables (i.e., moral competence, CPI-1, and CPYDS-2). Both groups had different initial status at the beginning (MC: *β* = .04, SE = .01, *P* < .01; CPI-1: *β* = .05, SE = .02, *P* < .01; CPYDS-2: *β* = .04, SE = .01, *P* < .01). Control group dropped slower (linear slope: MC = *β* = −.05, SE = .02, *P* < .01; CPI-1: *β* = −.06, SE = .03, *P* < .05; CPYDS-2: *β* = −.04, *SE* = .02, *P* < .05; cubic slope: MC = *β* = −.003, SE = .001, *P* < .01; CPI-1: *β* = −.01, SE = .002, *P* < .05; CPYDS-2: *β* = −.003, SE = .001, *P* < .01), but decelerated faster than did the experimental group (MC: *β* = .03, SE = .01, *P* < .01; CPI-1: *β* = .04, SE = .02, *P* < .05; CPYDS-2: *β* = .02, SE = .01, *P* < .05) across 8 waves. Similar trend that was also found in BC, except the test of group differences in linear slope was not significant (*β* = −.03, SE = .02, *P* > .05). These results revealed that both groups differed in their rates of growth over time and these differences occurred up through Wave 7 after which they diminished gradually (see Figures 1, 2, 3, and 4).

**Figure 5:**
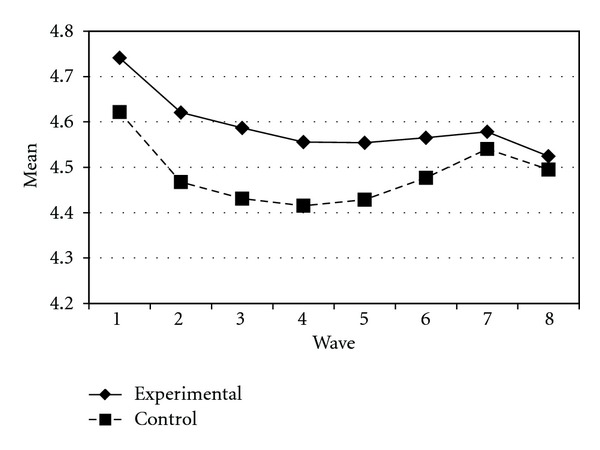
Growth trajectories of the experimental participants (experimental participants who regarded the program as beneficial) and control participants using PA (prosocial attributes second-order factor) as an outcome indicator.

**Figure 6:**
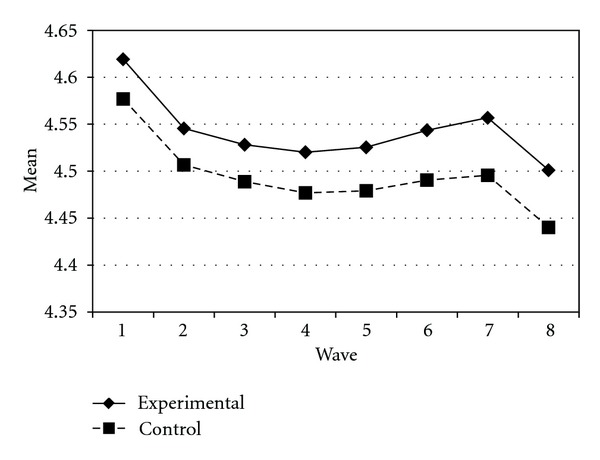
Growth trajectories of the experimental participants (experimental participants who regarded the program as beneficial) and control participants using PID ( positive identity second-order factor) as an outcome indicator.

**Figure 7:**
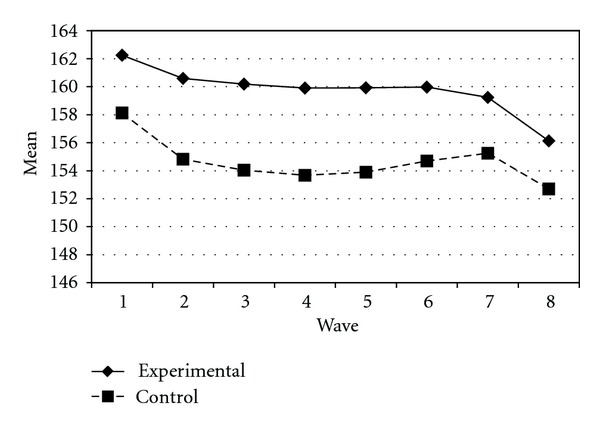
Growth trajectories of the experimental participants (experimental participants who regarded the program as beneficial) and control participants using KEY 36 (indicator based on 36 key items of the CPYDS) as an outcome indicator.

**Figure 8:**
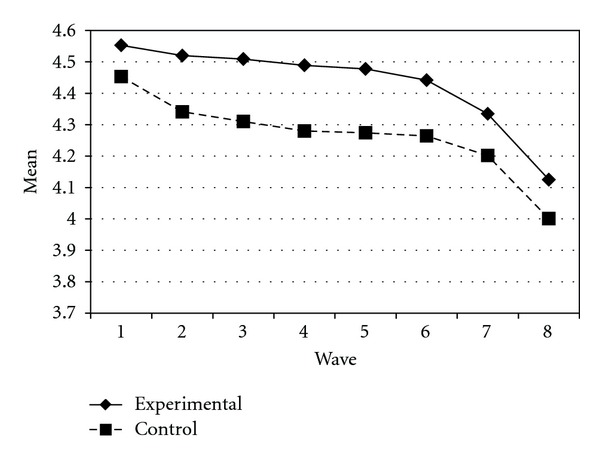
Growth trajectories of the experimental participants (experimental participants who regarded the program as beneficial) and control participants using BF (beliefs in the future subscale) as an outcome Indicator.

**Figure 9:**
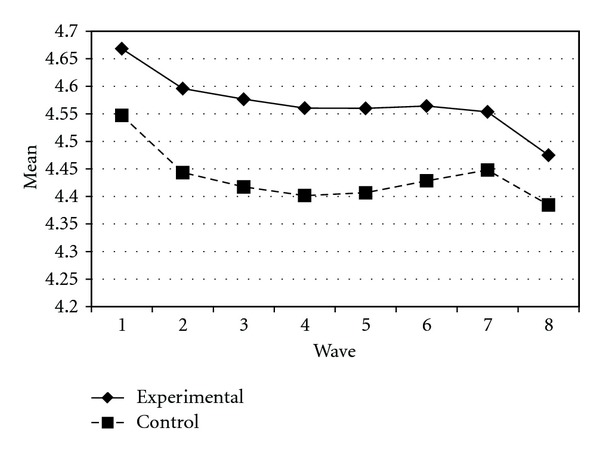
Growth trajectories of the experimental participants (experimental participants who regarded the program as beneficial) and control participants using CPYDS-12 (12 subscales of the CPYDS) as an outcome indicator.

**Figure 10:**
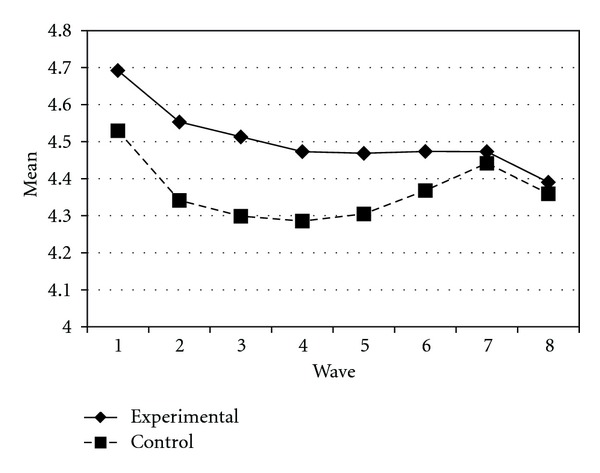
Growth trajectories of the experimental participants (experimental participants who regarded the program as beneficial) and control participants using PI (prosocial involvement subscale) as an outcome indicator.

**Figure 11:**
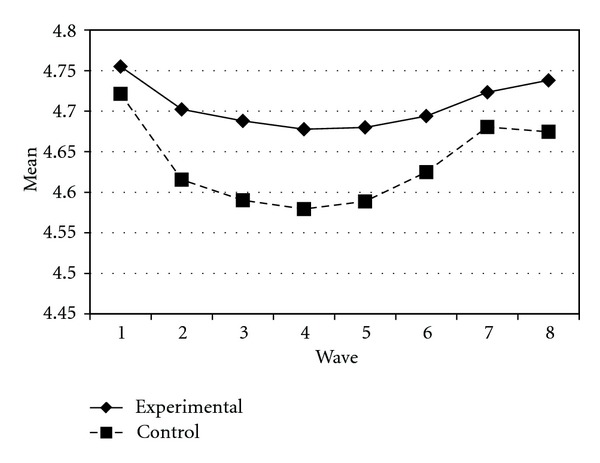
Growth trajectories of the experimental participants (experimental participants who regarded the program as beneficial) and control participants using PN-1 (one item from prosocial norm subscale) as an outcome indicator.

**Figure 12:**
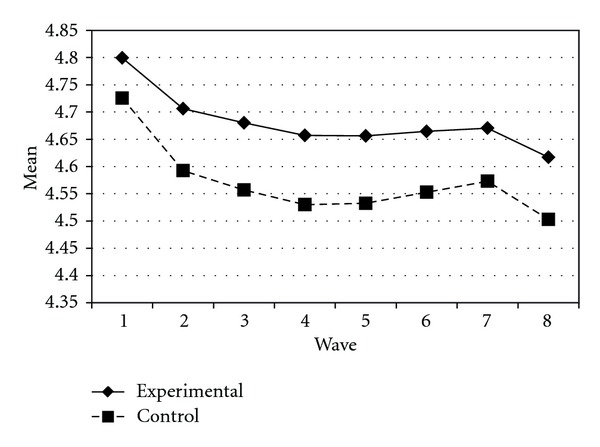
Growth trajectories of the experimental participants (experimental participants who regarded the program as beneficial) and control participants using RE (resilience subscale) as an outcome indicator.

Additional analyses were performed to examine the positive treatment effects by comparing the control group and experimental participants who found the program to be beneficial. More significant findings were shown in these analyses. Control group decreased faster and decelerated slower than did the experimental group (Tables 3 and 4). These patterns of change were shown in Figures 5, 6, 7, 8, 9, 10, 11, and 12. In general, these findings suggested that stable trajectories of positive youth development indicators were found in the experimental group, but not in the control group. These findings supported the beneficial treatment effect on participants' psychological development over time.

The values of ICC ranged from  .36 to  .64 (Tables 5 and 6), indicating the nested structure of the data [24, 25]. This also suggested that over 36% of the total variation in all variables was related to individual differences. To explore the effects of treatment on all outcome variables, the amount of variance in relation to the initial status and linear slope was examined. Based on the reduction of total variance from Model 1 (M1: baseline growth model) and Model 2 (M2: model with predictors only), treatment had stronger predictive effects in the within-individual variance, but lower in the between-individual variances. It is noteworthy that these results did not change much after entering the initial age and gender as covariates (Model 3: model with predictor and covariates) (Tables 5 and 6). Lastly, based on Feingold's [26] suggestions, the effect sizes of all IGC models were calculated. The effect sizes ranged from low to moderate (linear slope:  .00 to  .36; quadratic slope:  .00 to  .17; cubic slope:  .00 to  .02).

## 4. Discussion

Amongst different evaluation strategies, objective outcome evaluation is an important strategy. Objective outcome evaluation via randomized trials is also commonly regarded as the “gold standard” in establishing causal relationships. Despite its credibility, there are several problems of randomized trials. First, time is needed as longitudinal data are normally collected. Second, it is expensive because much manpower is involved in data collection over time. Third, attrition is a common problem in longitudinal evaluation studies. Nevertheless, experimental approach is still a widely used strategy to examine effectiveness of intervention programs. In the field of prevention, there are many examples of programs using trials to evaluate the treatment effects [27–30].

The purpose of this paper is to examine the effectiveness of a positive youth development program (Project P.A.T.H.S.) in Hong Kong by using a validated measure of positive youth development—the Chinese Positive Youth Development Scale. This is the first known scientific study that adopted a randomized group trial design using longitudinal data to evaluate a positive youth development program in the Chinese context. Consistent with previous longitudinal results [5–7, 31], participants from the experimental group generally performed better than those from the control group in terms of different positive youth development indicators. The experimental subjects, as compared to their control counterparts, had a more stable rate of growth among the three subscales of the CPYDS (i.e., moral competence, behavioral competence, and the CPYDS-2). These findings were further supported based on the experimental subjects who found the program to be beneficial to their development. The treatment effects on the linear, quadratic, and cubic slopes indicating the program successfully altered the trajectory of psychological development among adolescents [32]. It is noteworthy that this effect sustained even after the completion of the program for two years (i.e., Waves 7 and 8).

Recent analyses based on this cohort revealed that the positive impact of the Project P.A.T.H.S. on reducing problem and risk behaviors [9]. Given the paucity of positive youth development programs using strong experimental or quasiexperimental designs in Asian countries [4], this study provides the strongest evidence to date regarding the beneficial effects of the Project P.A.T.H.S. on improving adolescent psychological development. In conjunction with the existing findings [3, 33–37], the present study strongly suggest that the Project P.A.T.H.S. is able to promote positive development and reduce adolescent problem behavior in Chinese adolescents in Hong Kong.

In the present study, significant results that were more pronounced among those who perceived the program to be effective deserves some discussion. First, the findings suggest that positive youth development programs might not work in a “stimulus-response” manner and the cognitive appraisal of the participants is in fact important in determining the program outcome. As this factor is not properly addressed in the literature, it is recommended that further study should be carried out to examine how the subjective appraisal of the program participants might affect program outcomes through alteration of their motivation to join and participate. Second, the findings suggest that it is important to attend to the subjective outcomes perceived by the participants. As such, how to promote a sense of success and program ownership may help to promote program effectiveness in the long run.

Another significance of the study is the use of individual growth curve modeling to evaluate the impact of a positive youth program in a large sample across a five-year period, which is scarce in positive youth program literature. Researchers noted the needs of using advanced modeling techniques in longitudinal research [14, 38, 39]. Clearly, our study appears to be a positive response to this request.

There are several limitations in the present study. First, the data were based on self-report measures. Future research should evaluate the program by collecting longitudinal information from multiple methods (e.g., group interviews, diaries, and process evaluation) and sources (e.g., teachers, social workers, parents). Second, future research should examine the longitudinal effect of the positive youth development qualities on promoting psychological well-being (e.g., life satisfaction) and reducing problem behaviors (e.g., substance use, deliberate self-harm). This is supported by recent longitudinal findings which showed that positive youth development programs such as the Project P.A.T.H.S. can help to promote youth development and reduce their negative behavior among Hong Kong adolescents [8–10]. Despite the above limitations, the present study demonstrates the effectiveness of the Project P.A.T.H.S. in promoting positive youth development among Hong Kong adolescents. Basically, the study underscores the importance of designing positive youth programs for adolescents.

## Figures and Tables

**Table 1:** Number of collected questionnaires across waves.

*N* (Schools)	Wave 1	Wave 2	Wave 3	Wave 4	Wave 5	Wave 6	Wave 7	Wave 8
	48	47^a^	44^b^	44	43^c^	43	43	43
*N* (Participants)	7,846	7,388	6,939	6,697	6,876	6,733	6,116	5,934
Control Group	3,797	3,654	3,765	3,698	3,757	3,727	3,442	3,272
Male	1,936	1,876	1,896	1,888	1,874	1,894	1,770	1,663
Female	1,613	1,619	1,666	1,599	1,682	1,679	1,592	1,554
Experimental Group	4,049	3,734	3,174	2,999	3,119	3,006	2,674	2,662
Male	2,154	1,998	1,691	1,548	1,632	1,591	1,408	1,427
Female	1,745	1,571	1,283	1,259	1,312	1,278	1,155	1,191
% of successfully matched	98%	96%	97%	98%	99%	97%	93%	91%

^a^1 Experimental school (*n* = 207) had withdrawn after Wave 1.

^b^3 Experimental schools (*n* = 629) had withdrawn after Wave 2.

^c^1 Experimental school (*n* = 71) had withdrawn after Wave 4.

**Table 2:** Internal consistency and mean interitem correlations for all variables.

	Wave 1		Wave 2		Wave 3		Wave 4		Wave 5		Wave 6		Wave 7		Wave 8	
	*α*	Mean^a^	*α*	Mean^a^	*α*	Mean^a^	*α*	Mean^a^	*α*	Mean^a^	*α*	Mean^a^	*α*	Mean^a^	*α*	Mean^a^
BO	.83	.45	.85	.49	.86	.51	.88	.55	.88	.54	.88	.55	.86	.51	.87	.52
RE	.82	.44	.86	.50	.88	.54	.88	.55	.88	.55	.89	.56	.86	.51	.87	.52
SC	.83	.42	.86	.47	.87	.51	.88	.52	.87	.50	.89	.53	.87	.49	.87	.51
PB	.76	.44	.80	.51	.83	.55	.84	.58	.83	.56	.85	.58	.82	.54	.84	.57
EC	.83	.44	.85	.48	.86	.51	.86	.51	.86	.51	.87	.52	.85	.49	.86	.50
CC	.84	.47	.86	.52	.87	.54	.88	.55	.88	.54	.88	.56	.86	.52	.86	.52
BC	.76	.38	.80	.44	.82	.47	.83	.50	.82	.48	.83	.49	.81	.46	.81	.46
MC	.78	.37	.79	.39	.81	.42	.82	.43	.80	.41	.82	.44	.79	.39	.79	.39
SD	.76	.40	.80	.44	.82	.48	.82	.48	.81	.47	.82	.47	.80	.46	.81	.46
SE	.50	.34	.56	.39	.58	.41	.61	.44	.59	.42	.61	.43	.61	.44	.61	.44
CPI	.84	.43	.85	.45	.87	.48	.87	.49	.86	.47	.87	.48	.85	.46	.86	.46
BF	.82	.61	.83	.62	.84	.64	.84	.65	.84	.65	.85	.66	.79	.57	.81	.61
PI	.83	.49	.83	.50	.86	.55	.86	.54	.85	.52	.86	.55	.86	.54	.85	.54
PN	.77	.40	.80	.45	.81	.46	.81	.47	.81	.46	.81	.46	.82	.47	.81	.47
SP	.88	.51	.89	.56	.91	.60	.91	.62	.91	.60	.92	.62	.91	.61	.91	.61
KEY15	.88	.32	.89	.35	.90	.38	.90	.38	.90	.37	.90	.39	.89	.35	.89	.35
KEY36	.97	.32	.98	.34	.98	.37	.98	.37	.98	.36	.98	.38	.95	.36	.95	.36
CPYDS-2	.81	.38	.81	.39	.83	.42	.82	.40	.84	.43	.84	.43	.81	.39	.81	.38
CPYDS-10	.93	.56	.93	.59	.94	.61	.94	.62	.94	.61	.94	.62	.97	.36	.97	.36
CPYDS-12	.94	.56	.94	.56	.95	.59	.95	.58	.95	.58	.95	.60	.97	.34	.97	.34
CBC	.85	.66	.87	.69	.88	.71	.89	.72	.88	.71	.88	.72	.87	.69	.86	.68
PA	.79	.65	.77	.62	.79	.66	.77	.63	.78	.64	.79	.66	.79	.65	.74	.58
GPYDQ	.89	.52	.89	.53	.90	.55	.90	.55	.90	.54	.90	.57	.89	.53	.89	.52
PID	.83	.72	.84	.73	.85	.75	.86	.76	.85	.74	.86	.76	.84	.74	.84	.74

^a^Mean interitem correlation.

All parameters were significant (*P* < .05).

*Note*: BO: bonding; RE: resilience; SC: social competence; PB: recognition for positive behavior; EC: emotional competence; CC: cognitive competence; BC: behavioral competence; MC: moral competence; SD: self-determination; SE: self-efficacy; CPI: clear and positive identity; BF: beliefs in the future; PI: prosocial involvement; PN: prosocial norms; SP: spirituality; KEY15: indicator based on 15 key items of the CPYDS; KEY36: indicator based on 36 key items of the CPYDS; CPYDS-2: two subscales of the CPYDS; CPYDS-10: 10 subscales of the CPYDS; CPYDS-12: 12 subscales of the CPYDS; CBC: cognitive-behavioral competencies second-order factor; PA: prosocial attributes second-order factor; GPYDQ: general positive youth development qualities second-order factor; PID: positive identity second-order factor.

**Table 3:** Results of growth curve models for indicators derived from the CPYDS.

	Subjects joining the Tier 1 Program as experimental subjects				Subjects joining the Tier 1 Program and regarded the program as beneficial			
	MC	BC	CPI-1	CPYDS-2	PA	PID	KEY 36	BF
*Intercept*								
Initial status	4.50**	4.76**	4.45*	4.56**	4.68**	4.37**	160.23**	4.51**
Group	.04**	.04**	.05**	.04**	.06**	.06**	2.05**	.05**
Gender	.15**	.08**	.00	.15**	.13**	.02**	2.49**	.04**
Age	−.01	−.03**	−.02	−.02	−.05**	−.07	−1.29**	−.09**
*Linear slope*								
Initial status	−.17**	−.17**	−.15**	−.18**	−.28**	−.12**	−5.23**	−.15**
Group	−.05**	−.03	−.06*	−.04*	.04**	.06**	1.74**	.08**
Gender	−.08**	−.08**	−.09**	−.08**	−.09**	−.11**	−3.35**	−.11**
Age	.02	.02	.01	.02	.04**	−.05**	1.62**	.05*
*Quadratic slope*								
Initial status	.09**	.07**	.07**	.09**	.12**	.06**	2.46**	.06**
Group	.03**	.02*	.04*	.02*	−.03**	−.02**	−.82**	−.04**
Gender	.03**	.03**	.05**	.03**	.05**	.05**	1.41**	.05**
Age	−.01	.00	−.01	−.01	−.05	−.02*	−.71**	−.02
*Cubic slope*								
Initial status	−.01**	−.01**	−.01**	−.01**	−.02**	−.01**	−.35**	−.01**
Group	−.00**	−.00*	−.01*	−.00*	.00**	.00*	.09**	.00**
Gender	−.00**	.03**	−.01**	−.00	−.01**	−.01**	−.16**	−.01**
Age	.00	.00	.00	.00	.00	.00	.09*	.00

*Note*: MC = moral competence; BC = behavioral competence; CPI-1 = one item from clear and positive identity; CPYDS-2 = two subscales (i.e., behavioral competence and moral competence) of the CPYDS; PA = prosocial attributes second-order factor; PID = positive identity second-order factor; KEY 36 = indicator based on 36 key items of the CPYDS; BF = beliefs in the future.

*P* < .05*, *P* < .01**.

**Table 4:** Results of growth curve models for indicators derived from the CPYDS.

	Subjects joining the Tier 1 Program and regarded the Program as Beneficial			
	CPYDS-12	PI	PN-1	RE
*Intercept*				
Initial status	4.61**	4.61**	4.74**	4.76**
Group	.06**	.08**	.02	.04**
Gender	.08**	.10**	.13**	.05**
Age	−.04**	−.06**	−.04**	−.01
*Linear slope*				
Initial status	−.18**	−.33**	−.16**	−.23**
Group	.03**	.06**	.06*	.04**
Gender	−.09**	−.09**	−.11**	−.07**
Age	.04**	.06**	−.11**	.05**
*Quadratic slope*				
Initial status	.08**	.15**	.07**	.10**
Group	−.02**	−.04**	−.03*	−.02*
Gender	.04**	.05**	.05**	.02**
Age	−.02*	−.02*	−.01	−.03**
*Cubic slope*				
Initial status	−.01**	−.02**	−.01**	−.01**
Group	.00**	.01**	.00	.00*
Gender	−.00**	−.01**	−.01**	−.00*
Age	.00*	.00*	.00	.00*

*Note*: CPYDS-12: 12 subscales of the CPYDS; PI: prosocial involvement; PN-1: one item from prosocial norms; RE: resilience.

*P* < .05*, *P* < .01**.

**Table 5:** Results of intraclass correlation coefficients and within- and between-individual variances from the CPYDS.

	Subjects joining the Tier 1 Program as experimental subjects				Subjects joining the Tier 1 Program and regarded the program as beneficial			
	MC	BC	CPI-1	CPYDS-2	PA	PID	KEY 36	BF
Within-individual variance
M1	.29	.30	.79	.27	.28	.32	207.55	.50
M2	.27	.28	.75	.24	.25	.29	184.59	.46
M3	.27	.28	.75	.24	.25	.29	183.78	.46
Between-individual variance
* Intercept*								
M1	.30	.26	.51	.29	.34	.47	429.71	.59
M2	.28	.25	.51	.28	.29	.45	397.11	.56
M3	.27	.25	.50	.27	.28	.44	396.11	.55
* Linear slope*								
M1	.00	.00	.00	.00	.01	.01	9.74	.01
M2	.01	.00	.01	.01	.01	.01	11.81	.02
M3	.01	.00	.01	.01	.01	.01	11.82	.02
Baseline model								
* Within-individual*	.31	.31	.81	.28	.30	.36	235.33	.55
* Between-individual*	.30	.27	.52	.29	.33	.47	420.26	.59
ICC	.64	.46	.39	.51	.53	.57	.64	.52

*Note*: MC: moral competence; BC: behavioral competence; CPI-1: one item from clear and positive identity; CPYDS-2: two subscales (i.e., behavioral competence and moral competence) of the CPYDS; PA: prosocial attributes second-order factor; PID: positive identity second-order factor; KEY 36: indicator based on 36 key items of the CPYDS; BF: beliefs in the future.

M1: baseline growth model. M2: predictors only model. M3: predictors and covariates model. ICC: intraclass correlation coefficients.

*P* < .05*, *P* < .01**.

**Table 6:** Results of intraclass correlation coefficients and within- and between-individual variances from the CPYDS.

	Subjects joining the Tier 1 Program and regarded the program as beneficial			
	CPYDS-12	PI	PN-1	RE
Within-individual variance
M1	.15	.74	.74	.32
M2	.14	.69	.69	.29
M3	.14	.69	.69	.29
Between-individual variance
* Intercept*				
M1	.32	.41	.41	.35
M2	.29	.38	.38	.30
M3	.28	.37	.37	.30
*Linear slope *				
M1	.01	.00	.00	.01
M2	.01	.01	.01	.01
M3	.01	.01	.01	.01
Baseline model				
*Within-individual *	.17	.44	.75	.34
*Between-individual *	.31	.37	.42	.35
ICC	.64	.45	.36	.51

*Note*: CPYDS-12: 12 subscales of the CPYDS; PI: prosocial involvement; PN-1: one item from prosocial norms; RE: resilience.

M1: baseline growth model. M2: predictors only model. M3: predictors and covariates model. ICC: intraclass correlation coefficients.

*P* < .05*, *P* < .01**.
